# Temporal-Spatial Feature Extraction of DSA Video and Its Application in AVM Diagnosis

**DOI:** 10.3389/fneur.2021.655523

**Published:** 2021-05-28

**Authors:** Keke Shi, Weiping Xiao, Guoqing Wu, Yang Xiao, Yu Lei, Jinhua Yu, Yuxiang Gu

**Affiliations:** ^1^Department of Electronic Engineering, Fudan University, Shanghai, China; ^2^Shenzhen Institutes of Advanced Technology, Chinese Academy of Sciences, Shenzhen, China; ^3^Department of Neurosurgery, Huashan Hospital, Fudan University, Shanghai, China; ^4^School of Information Science and Technology, Fudan University, Shanghai, China; ^5^Shanghai Key Laboratory of Brain Function Restoration and Neural Regeneration, Huashan Hospital, Shanghai, China

**Keywords:** radiomics approach, real-time image, temporal features, Faster-RCNN, digital subtraction angiography, arteriovenous malformation

## Abstract

**Objectives:** Brain arteriovenous malformation (AVM) is one of the most common causes of intracranial hemorrhage in young adults, and its expeditious diagnosis on digital subtraction angiography (DSA) is essential for clinical decision-making. This paper firstly proposed a deep learning network to extract vascular time-domain features from DSA videos. Then, the temporal features were combined with spatial radiomics features to build an AVM-assisted diagnosis model.

**Materials and method:** Anteroposterior position (AP) DSA videos from 305 patients, 153 normal and 152 with AVM, were analyzed. A deep learning network based on Faster-RCNN was proposed to track important vascular features in DSA. Then the appearance order of important vascular structures was quantified as the temporal features. The structure distribution and morphological features of vessels were quantified as 1,750 radiomics features. Temporal features and radiomics features were fused in a classifier based on sparse representation and support vector machine. An AVM diagnosis and grading system that combined the temporal and spatial radiomics features of DSA was finally proposed. Accuracy (ACC), sensitivity (SENS), specificity (SPEC), and area under the receiver operating characteristic curve (AUC) were calculated to evaluate the performance of the radiomics model.

**Results:** For cerebrovascular structure detection, the average precision (AP) was 0.922, 0.991, 0.769, 0.899, and 0.929 for internal carotid artery, Willis circle, vessels, large veins, and venous sinuses, respectively. The mean average precision (mAP) of five time phases was 0.902. For AVM diagnosis, the models based on temporal features, radiomics features, and combined features achieved AUC of 0.916, 0.918, and 0.942, respectively. In the AVM grading task, the proposed combined model also achieved AUC of 0.871 in the independent testing set.

**Conclusion:** DSA videos provide rich temporal and spatial distribution characteristics of cerebral blood vessels. Clinicians often interpret these features based on subjective experience. This paper proposes a scheme based on deep learning and traditional machine learning, which effectively integrates the complex spatiotemporal features in DSA, and verifies the value of this scheme in the diagnosis of AVM.

## Introduction

Digital subtraction angiography (DSA) is the most important imaging technique for cerebrovascular disease diagnosis (1). As a type of real-time images, it provides a unique combination of high spatial and temporal resolution, and it is able to exquisitely delineate the location and number of feeding vessels and the pattern of perfusion (2). Brain arteriovenous malformations (AVM) are fast-flow vascular networks characterized by direct shunts from feeding arteries to draining veins, devoid of the interposed capillary system. AVM is more common in the central nervous system (CNS) than other organs, with a prevalence rate of 1.3 per 100,000 population (3). Although only a small portion of brain AVM patients present symptoms of headache or seizure, a morbidity rate of 30–50% and a mortality rate of 10–15% were reported in young adults with intracerebral hemorrhage (4). The definitive diagnosis of AVM relies on cerebral DSA (2). However, it might be labor- and time-consuming for inexperienced centers or doctors to read a 2D-DSA video and give an accurate diagnosis of brain AVM. Therefore, an automatic AVM diagnosis system would be helpful to provide objective diagnostic hints, especially in emergency cases.

Recent studies on AVM diagnosis have focused on AVM nidus extraction and vessel classification based on 4D CTA (computed tomography angiography) or 3DRA (three-dimensional rotational angiography) medical images (5–7) and focus on the extraction of AVM nidus and the classification of feeding vessels and drain vessels (8). However, these images are not able to provide information of vascular distribution and perfusion. DSA is the gold standard of cerebrovascular diagnosis that contains all the information mentioned above, but it is difficult to extract information from a DSA video since no effective method has been developed yet. Our study proposed a methodology to extract vascular distribution and perfusion from a DSA video in an automated manner by combining Faster-RCNN and radiomics method, where Faster-RCNN is used to detect vessel structures and radiomics is used to obtain static image features.

Faster-RCNN is an algorithm, and it used to recognize the blood vessels of a DSA video in our method. It is widely used in target detection and recognition in natural images (9–12) and also shows high efficiency in clinical applications. It has been used in the region of interest detection and lesion localization on medical images, such as ultrasound images, X-ray images, and CT images (13–15). Sa et al. (13) applied a fine-tuned Faster-RCNN trained on natural images to identify landmark points in lateral lumbar X-ray images and demonstrated that using very small annotated clinical datasets can also achieve great accuracy. Radiomics is used to transfer medical images into high-dimensional, mineable features that reflect underlying pathophysiological information (16) and has a great potential in precise diagnosis and treatment planning. It uses machine learning or deep learning techniques to solve various clinical tasks (17).

Automated AVM diagnosis is helpful to make the diagnosis of AVM more objective and reliable. Designing an automated AVM diagnosis model based on a DSA video is a challenging task because of the following obstacles. First of all, most of the existing medical image diagnosis models are based on static images. DSA reflects the perfusion of a three-dimensional vascular network that changes with time. The existing modeling methods based on static images are not suitable. Secondly, in DSA imaging, the sequence of vascular appearing reflects differences in vascular perfusion, and these differences are essential for the diagnosis of cerebrovascular diseases. Because of the complexity of the cerebrovascular network, it is difficult to quantitatively evaluate the sequence of key blood vessels. Thirdly, the assessment of images depends on the experience of doctors, and inconsistencies of the results among different doctors for the same case are likely to occur.

To overcome these challenges, an automatic detection model of the blood vessel phase based on Fast-RCNN is proposed. This model can automatically identify the early arterial phase, the late arterial phase, the early venous phase, the late venous phase with one sinus, and the late venous phase of the cerebral vessels. According to the results of the time phase detection, the time characteristics contained in the DSA image can be obtained. Then the key frames in the DSA image are extracted to calculate the radiomics features. Finally, the temporal features and radiomics features are fused to establish the final AVM diagnostic model.

## Materials and Methods

### Patients and Materials

This study protocol was approved by the ethics committees of the Huashan Hospital, Fudan University, and informed consent was waived since the retrospective study. From January 2010 to December 2013, 1,025 patients with cerebrovascular diseases who underwent DSA examination before operation or conservative treatment were reviewed. All 2D-DSA were conducted by senior neurosurgeons or neuro-interventional radiologists with more than 10 years of experience, on a Philips UNIQ FD20 digital subtraction biplane angiographic system. After a sheath (with an internal diameter of 1.65 mm and 10 cm long) was placed inside the femoral artery and an angiopointer (with an internal diameter of 1.22 mm and length of 100 cm) was placed at the beginning of ICA or VA, the contrast agent was injected by a contrast delivery system (Angiomat Illumena). During the 6-s anteroposterior position digital subtraction angiography image acquisition, 6 ml of the contrast agent was injected at a rate of 4 ml/s under a pressure of 200 Pa to obtain frames, where rates of Philips' instrument varied from 166 to 333 ms/frame.

AVM cases that are included in this study must satisfy the following aspects: (1) cases have anteroposterior position 2D-DSA videos; (2) lesions are visible in anteroposterior position images by experienced doctors; and (3) AVM cases without other diseases such as moyamoya disease and brain tumor (18), which may bias the DSA video and cause uncertainty in the validation of our model. The inclusion criteria for non-AVN cases in this study are as follows: (1) cases show negative results in the DSA video but are diagnosed by other medical image and (2) blood vessel disease cases that do not affect the distribution of blood vessels, such as aneurysm.

Finally, a total of 305 cases (153 non-AVM vs. 152 AVM) were collected. Among the 153 non-AVM cases, 31 were diagnosed with cavernous hemangioma by MRI, and 46 were diagnosed as aneurysm-negative by DSA, but confirmed as spontaneous subarachnoid hemorrhage in CT. Thirty-six cases were negative in DSA anteroposterior position images but were confirmed with aneurysms in three-dimensional rotational angiography (3DRA). Forty cases were suspected of aneurysms by computed tomography angiography (CTA) or magnetic resonance angiography (MRA) but proved to be normal or artery ectasia by DSA. Table 1 shows information about the age, gender, and Martin-Spetzler Score (19) of the AVM patients.

**Table 1:** Baseline characteristics of patients with lower levels of brain AVM (Grade I,II,III) and higher levels of brain AVM (Grade IV, V).

**Variables**	**Grade I, II, III**	**Grade IV, V**	***p*-value**
Gender(M/F)	81 (43/38)	71 (37/34)	0.977
Age	29.12 ± 13.57	28.87 ± 12.43	0.899
Smoking			0.073
Non-smoking	57 (70.3%)	58 (82.8%)	
Smoking	24 (29.7%)	13 (17.2%)	
Drinking			0.315
Non-drinking	63 (77.8%)	59(84.2%)	
Drinking	18 (22.2%)	12 (16.8%)	
Hypertension			0.951
Non-hypertension	72 (88.9%)	62 (88.5%)	
Hypertension	9 (11.1%)	9 (11.5%)	
Size			<0.001
Small (<3 cm)	48 (59.2%)	0	
Medium (3–6 cm)	33 (40.8%)	44 (61.9%)	
Large (>6 cm)	0	27 (38.1%)	
Location	<0.001
Non-situated in neurological	13 (16.0%)	1 (1.40%)	
Critical areas			
Situated in neurological	68 (84.0%)	70 (98.6%)	
Critical areas			
Deep venous drainage			<0.001
Non-deep venous drainage	26 (32.1%)	7 (9.80%)	
Deep venous drainage	55 (67.9%)	64 (90.2%)	

### Feature Extraction and Selection

#### Vascular Structure Detection

**Figure 1:**
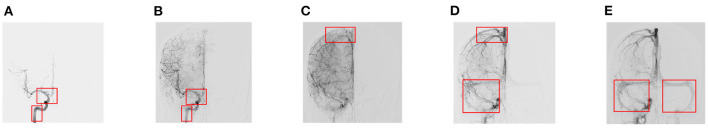
Annotation details of different phases. **(A)** Early arterial phase. **(B)** Late arterial phase. **(C)** Early venous phase. **(D)** Late venous phase with one sinus. **(E)** Late venous phase with two sinuses.

AVM will change the perfusion characteristics of the patient's cerebrovascular network. In a DSA video, doctors mainly diagnose AVM and judge its severity based on the sequence of appearance of the main blood vessels and the structural characteristics of the vascular network. Because of the complex structure of cerebral blood vessels, it is difficult for the human eye to accurately quantify the order of appearance of the main blood vessels. We conducted a target detection algorithm, Faster-RCNN, to obtain DSA sequence information. Two specialists, who have been engaged in clinical cerebrovascular disease for more than 5 years, annotated the vascular structures in different phases, as shown in Figure 1. If there were any ambiguity, the third senior doctor would review and give the final decision.

**Figure 2:**
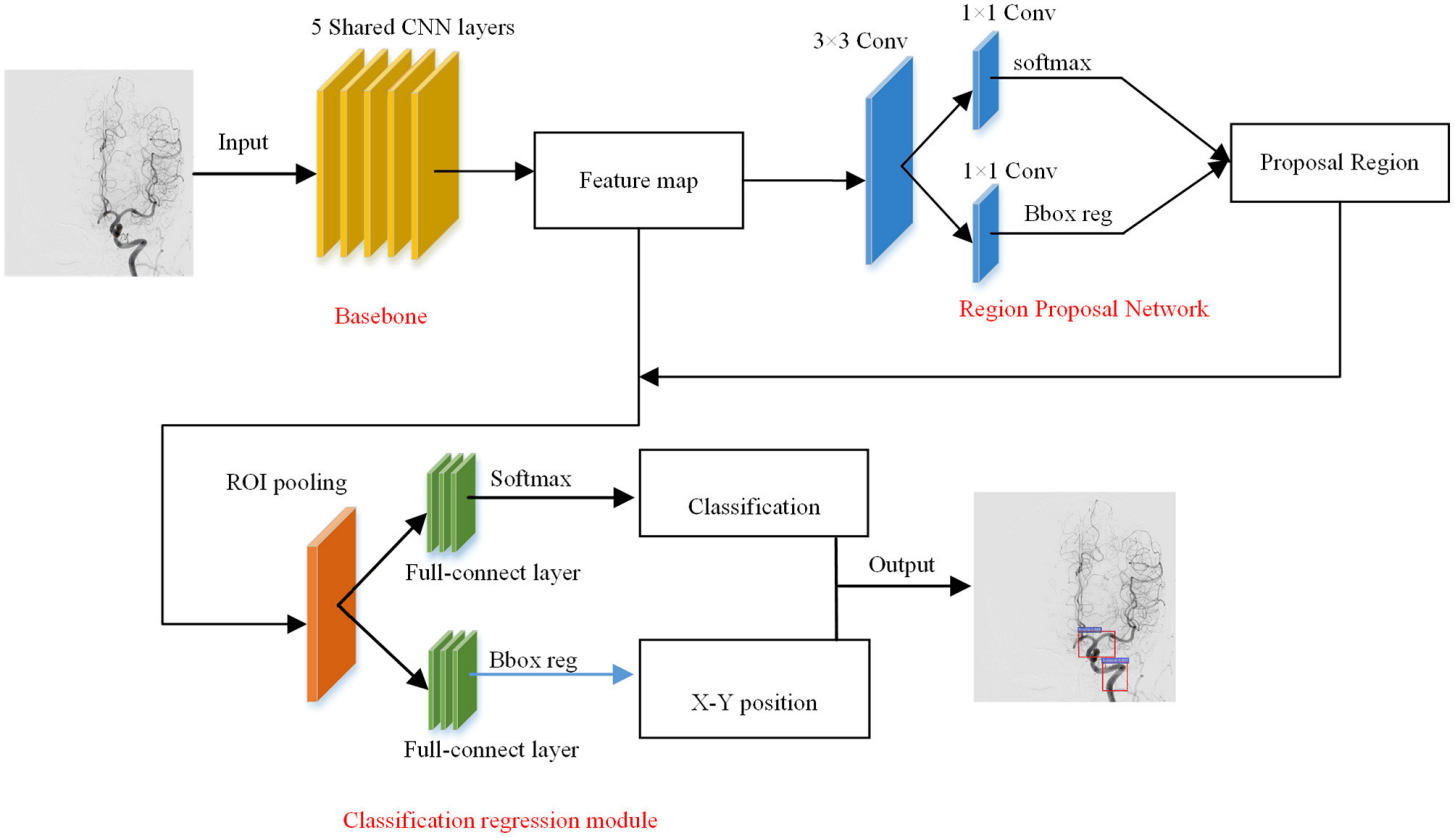
The structure of Faster-RCNN network.

In the vessel structure recognition, we have annotated the key vessels in the DSA video. As shown in Figure 1, rectangular boxes of different sizes are used to mark important vascular structures such as carotid artery, Willis circle, large vein, venous vessel, and venous sinus. In 153 non-AVM patients, a total of 1,714 vascular structures were annotated, 80% of which were used for training of the Faster-RCNN network (Figure 2) and 20% were used for testing. The labeling statistics of each vascular structure are shown in Table 2. We applied average precision (AP), mean average precision (mAP), and intersection-over-union (IoU) to evaluate the performance of the detection model. The AP of each cerebrovascular structure and the mAP on the test set were calculated to evaluate the performance of the multivessel detection model.

**Table 2:** Data summary of structures annotations.

	**Category**	**Training set**	**Test set**	**Total**
Annotation num	Carotid artery	260	62	322
	Willis circle	267	67	334
	Vein	280	70	350
	Venous Vessel	276	70	346
	Venous sinus	289	73	362
	Total	1,372	342	1,714

#### Temporal Features

The order in which important blood vessels appear defines the different phases of the DSA video (Table 3). According to the results of Fast-RCNN automatic tracking, DSA videos are divided into five phases: early arterial phase, late arterial phase, capillary phase, early venous phase, and late venous phase, respectively, according to the criteria described in Table 3. Each phase can be further divided into early and later phases. In order to facilitate computer quantification, complex blood vessel temporal information is integrated into five temporal features. These five characteristics can simply and directly describe the sequence of different time phases. Details of the proposed five temporal features were summarized in Table 3.

**Table 3:** Phase classification and temporal features extraction.

	**Phase classification**	
Early arterial phase	Both early and late arterial phases only detect the internal carotid artery and the circle of Willis. The vascular tree in early phase is incomplete, and it is complete in late phase.	
Late arterial phase	
Capillary phase	There is no apparent blood vessel.	
Early venous	This phase only has large veins.	
Late venous	This phase has both large veins and sinuses	
	**Temporal features**	**Features value**
T1	Venous sinuses appear before the end of the artery.	Yes (1) No (0)
T2	Venous sinuses appeared before the disappearance of the circle of Willis.	Yes (1) No (0)
T3	Venous sinuses appear before venous vessels.	Yes (1) No (0)
T4	Veins appear before wills ring disappeared.	Yes (1) No (0)
T5	No obvious capillary phase.	Yes (1) No (0)

#### Radiomics Features

In addition to the temporal feature, the distribution structure of the vascular network is also an important feature for AVM diagnosis (20). Therefore, in this step, we use the radiomics method to extract the radiomics features in the DSA static frames. To reduce the subjectivity of data selection, we selected five frames in equal proportion from the beginning to the end of a video. Radiomics features were extracted from these five DSA frames. Obtained features mainly fell into three groups: intensity, texture, and wavelets (21–23). The intensity group consisted of 16 features that describe the overall intensity and heterogeneity information of the whole image. The texture group contained 54 features, estimating the gray-level regional spatial distribution. In the wavelet group, we transformed the intensity and texture features into eight frequency subbands *via* wavelets to obtain additional information, obtaining 280 features.

#### Feature Integration

To verify the significance of the proposed features, three sets of features were determined:

*i The temporal features*. Table 3 shows the detailed information of the temporal features. This group of features was set to determine the association between temporal features and AVM diagnosis.
*ii The radiomics features*. A total of 1,750 (350 × 5) radiomics features were extracted from each sample. This group of features was set to represent the vessel distribution characteristics for AVM diagnosis.
*iii The combined features*. These two feature sets were concatenated into an integrative dataset, and a model was expected from two types of features with higher accuracy that should capture static and dynamic information from input images.

To exclude redundant and irrelevant features, we applied the iterative sparse representation (ISR) for feature selection (24, 25). A partial sample was selected for SR in each iteration, and the result of multiple SRs was calculated on average to obtain coefficients, denoting the importance of the corresponding feature.

### Classification

We applied the support vector machine (SVM) as our classifier, which is efficient in machine-learning tasks with limited samples (26–28).

For AVM diagnosis, 305 cases were randomly divided into two groups: an independent testing cohort and a cross-validation cohort at a ratio of 3–7. We also applied the leave-one-out (LOO) cross-validation test diagnosis model with different feature sets. Then, the independent validation set was used for further evaluation of the diagnostic performance of the diagnosis model. We calculated the area receiver operating characteristic (ROC) curve to establish the overall performance of the models.

For AVM grading, a total of 152 AP series with visible nidus were considered as cross-validation cohorts, shown in Table 1. Accuracy (ACC), sensitivity (SENS), specific (SPEC), and area under the ROC (AUC) are used to evaluate the performance of our model.

**Figure 3:**
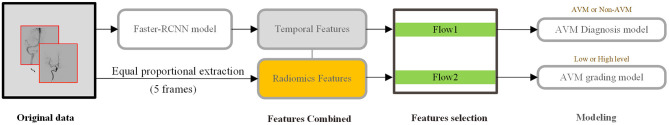
DSA analysis process from extraction to model building. Temporal features are obtained from the whole series, while radiomics features are extracted from 5 frames, representing early arterial phase, later arterial phase, capillary phase and vein phase, and sinus phase.

The overall flowchart was shown in Figure 3. The injections for fine-tuned Faster-RCNN were complete DSA sequences, which were used to obtain temporal features. For diagnosis modeling, the final input were radiomics features collected from our equal-proportional sampling and temporal features obtained from the Faster-RCNN model.

## Results

### The Cerebrovascular Structure Detection

**Figure 4:**
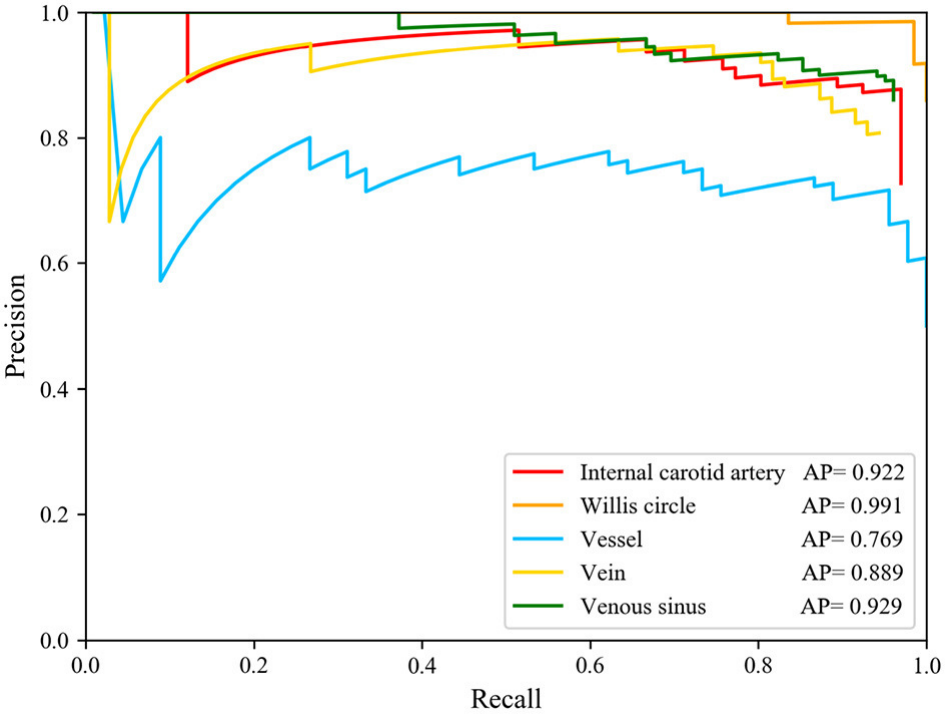
Precision-Recall curve of five structure and AP value of each structures. The AP of the internal carotid artery is 0.922, of the circle of Willis is 0.991, of the vein is 0.889, of the venous sinus is 0.929, and of the venous blood vessel is 0.769.

Based on the predicted results, we calculated the Precision–Recall (P-R) curve of five types of blood vessel ROI and calculated the area under the P-R curve (average precision) to measure the model's detection precision of each blood vessel structure. We used the target detection model to calculate the AP values of the internal carotid artery, the Willis circle, the large vein, the venous blood vessel, and the venous sinus, respectively. The results are shown in Figure 4. In the test set, the AP of the vein was 0.889, that of the internal carotid artery was 0.922, that of the circle of Willis was 0.991, that of the venous sinus was 0.929, and that of the venous blood vessel was 0.769.

**Figure 5:**
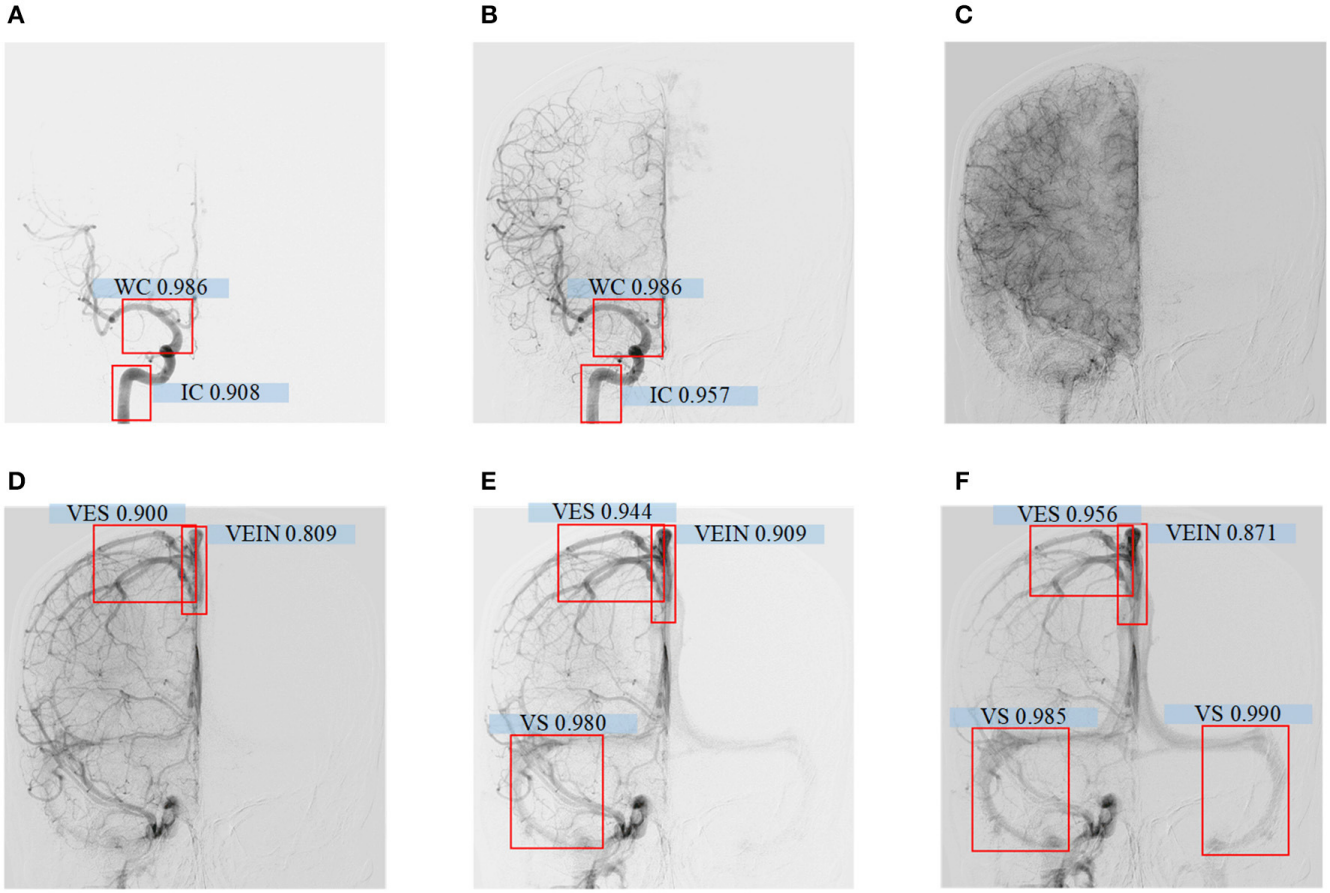
**(A–F)** Detecting results of DSA videos with different phase. From left to right are arterial phase I, II, capillary phase, vein phase, venous sinus phase I, and venous sinus phase II. In arterial phase I, the confidence level of the internal carotid artery is 0.908, the Willis Circle is 0.986; In arterial phase II, the internal carotid artery is 0.957, the Wil lis Circle is 0.986; In vein phase, the confidence level of vein detection is 0.809, venous vessel is 0.900; In venous sinus phase I, the confidence level of vein is 0.909, venous vessel is 0.944, venous sinus is 0.980; In venous sinus phase II, the confid ence level of large vein is 0.871, venous vessel detection is 0.956, and venous sinus is 0.985 and 0.990.

The model had good performance in discriminating the circle of Willis and deficient in discriminating veins. The mAP of the five types of blood vessel, detected by this model, was 0.902. We can conclude that this model had good performance in the detection of vascular structure based on DSA. Then, we visualized the detection results of the representative images. The model can detect the positions of the internal carotid artery and the circle of Willis in the arterial phase images, as shown in Figures 5A,B. The positions of large veins, venous blood vessels, and venous sinuses can be detected in venous phase images, as shown in Figures 5D–F. The capillary phase is shown in Figure 5C, where no obvious vascular structure can be detected.

### AVM Diagnosis

**Figure 6:**
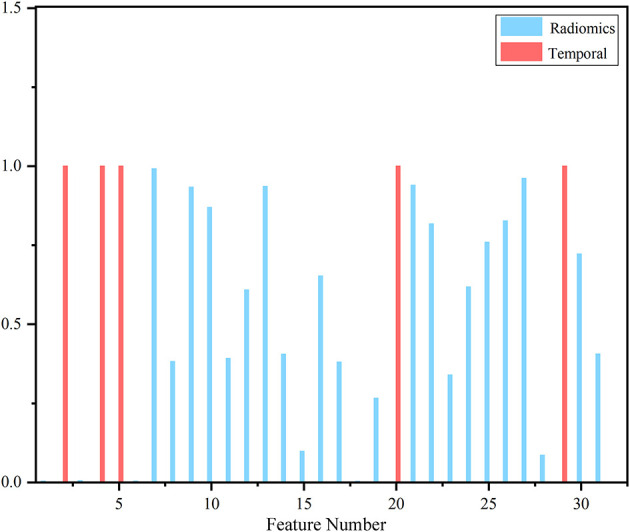
Contribution of selected features. The red bar represented temporal features, and the blue bar corresponded to traditional static image featu res that clearly showed temporal features took precedence over most radiomics features.

**Figure 7:**
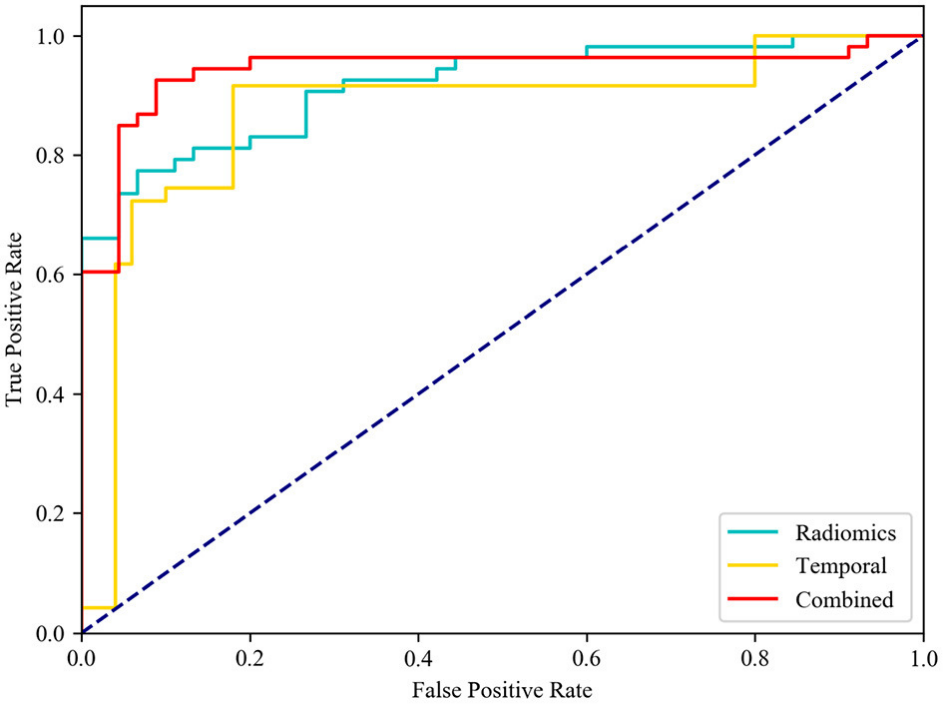
Receiver operating characteristic (ROC) curves. Diagnostic performance of different feature group models in independent testing. While comparing the AUC curve s between radiomics, temporal and combined features, the AUC of combined features [0.942, (0.875–0.979)] was the highest one.

After feature selection, the feature contribution of the combined features is shown in Figure 6. Table 4 illustrates the classification results by LOO cross-validation and independent validation cohorts in three different feature sets. The corresponding model was firstly determined in an LOO cross-validation experiment based on AUC. Then, we evaluated the model on the independent validation set. The traditional radiomics method achieved an ACC of 0.856 and an AUC of 0.913. The temporal features obtained an ACC of 0.850 and an AUC of 0.873. Our proposed method (combined features) yielded an ACC of 0.889 and an AUC of 0.967 in differentiating between AVM and non-AVM DSA (Table 4). Receiver operating characteristic (ROC) curves of the three features group are summarized in Figure 7.

**Table 4:** Performance comparison of the model trained by different feature sets.

	**Feature set**	**AUC (0.95 CI)**	**ACC**	**SENS**	**SPEC**
LOO cross-validation cohort	Radiomics	0.956 (0.929~0.976)	0.883	0.843	0.929
	Temporal	0.877 (0.837~0.911)	0.865	0.849	0.884
	Combined	0.971 (0.947~0.986)	0.937	0.911	0.967
Independent testing cohort	Radiomics	0.918 (0.845~0.963)	0.856	0.856	0.855
	Temporal	0.916 (0.843~0.963)	0.836	0.735	0.935
	Combined	0.942 (0.875~0.979)	0.889	0.943	0.823

### AVM Grading

As shown in Table 1, age, smoking history, and hypertension cases were significantly different between the normal cases and the brain AVM patients. The baseline characteristics of patients with lower levels of brain AVM (Grade I, II, III) and higher levels of brain AVM (Grade IV, V) are presented in Table 1. More patients with higher levels of brain AVM (Grade IV, V) presented with epilepsy than patients with lower levels of brain AVM (Grade I, II, III) (*p* <0.001), probably due to the stimulus of larger nidus to the cortex.

Considering the small number of cases and the imbalance of data at all scores, we were able to design both high (Grade IV, V) and low (Grade I, II, III) level classification tasks. To verify the effectiveness of temporal features, combined, and radiomics features were considered in this task. The model's indicators with and without temporal features are shown in Table 5. After combining the temporal features, the indicator had a slight increase in accuracy. This result proved that the temporal features had an effect on grading of the deformity group.

**Table 5:** Performance comparation between two models.

**#**	**Feature set**	**AUC (0.95 CI)**	**ACC**	**SPEC**	**SENS**
1	Combined	0.871(0.813~0.916)	0.840	0.866	0.797
2	Radiomics	0.863(0.807~0.911)	0.806	0.812	0.796

## Discussion

Being relatively convenient and non-invasive, CTA and MRA usually serve as the primary tools for screening brain AVM after patients suffer from headache or seizure. Depending on postprocessing, CTA and MRA often demonstrate insufficient resolution and artifacts. Moreover, CTA and MRA could only reveal the whole brain vessels in a static image. Therefore, they cannot be used to accurately evaluate AVM. In contrast, through contrast injection into one single brain inflow artery, DSA is able to clearly delineate the feeding and draining vessels of AVM. The primary diagnosis of AVM needs to be ultimately confirmed by DSA. In addition, one of the classic grading systems on brain AVM, the Spetzler–Martin (SM) (19) grade could be scored based on DSA videos with regards to the size, the pattern of venous drainage, and the neurological eloquence of the adjacent brain (18, 19). This could provide guidance in further treatment decision-making. Specifically, multimodality approaches by microsurgical resection, endovascular embolization, and radiosurgical technique could be applied to Grades I, II, and III brain AVMs, while the recommended management of Grades IV and V brain AVMs is conservative treatment (19, 29). Therefore, patients suspected of brain AVM are expected to undergo DSA examination, regardless of the following varied treatment techniques. It might be challenging for junior doctors or inexperienced ones to give a definitive diagnosis. The model we built up using vascular phase feature extraction based on deep learning and DSA radiomics features based on machine learning is able to expeditiously recognize brain AVM on anteroposterior position DSA videos, shedding lights on brain AVM artificial intelligence study.

The DSA video provides both dynamic information and static information, which is valuable for cerebrovascular disease diagnosis. The characteristics of DSA imaging are as follows: as the contrast medium flows in the cerebrovascular, the developed structure will gradually change, and the number of frames in each case is unpredictable (30). DSA video images and natural video images have similarities that they can reflect the state of the target within a certain time period, but the difference is that natural video images are usually RGB images, reflecting the behavior of objects, while the DSA videos are grayscale, reflecting the periodic changes of the cerebrovascular structure with limited and unfixed frames. Most researches focused on how to extract features from the video that can describe the video actions better. The traditional video classification usually uses static apparent and temporal features for classification tasks. The preferred temporal features include spatial–temporal interest points, the histogram of the optical flow, dense trajectories (31–34), and so on. The above features are manually designed features and require lots of prior knowledge. Therefore, video classification based on deep learning is favored by more and more researchers and has become the mainstream research method in this field. The preferred methods are the dual-stream method (35), using CNN to extract spatial features and optical flow to obtain temporal features, and long-term recurrent convolution (LRCN) (36, 37), which extracts the apparent information of the video frame through the CNN layer and retains the information of time dimension through the LSTM layer.

However, these classic video classification methods are not suitable for DSA videos for three reasons: First, the number of DSA frames is not fixed, which is usually between 20 and 50 frames. The development status of each frame is related to the patient's blood flow rate and the shooting interval of the machine. In other words, when random frames from each case are selected, the change of the image in time dimension will be different. Second, there are interference, noise, and artifacts that will influence the image quality. Therefore, it is difficult to obtain effective information. Finally, the number of DSA data is limited in our research, which makes it hard to meet the conditions of deep learning.

The Faster-RCNN model achieves good performance in vessel structure detection, and the temporal feature, which was introduced in this study, provides the temporal features of the real-time image. We can use Faster-RCNN to obtain reliable temporal features and eliminate the undeveloped frame automatically. First, from the beginning to the end of the development frame, five frames are selected in equal proportions, representing the pre-arterial phase, the late arterial phase, the capillary phase, the early vein, and the sinus phase, respectively. Then, we extract image features in selected frames. Finally, two types of features are combined to train the SVM model. It is obvious that a better performance classifier can be achieved if we manually select the frame that contains the biggest nidus and annotate the ROI area to make training datasets, or even collect the time sequence features through human eyes. However, this method increases the burden of the medical staff, violates the original intention of automatic diagnosis, and cannot be applied to clinical diagnosis. In our proposed method, although the Faster-RCNN may make mistakes occasionally in the structure detection of abnormal cases, we take the structure detection results of all frames into consideration when calculating the temporal features to avoid those mistakes. Therefore, if one or two frames are detected incorrectly during the whole process, it will not affect the final temporal features. Based on the reasons above, there is no external intervention in the acquisition of static image features and temporal features in the proposed method.

In our study of AVM diagnosis, the model trained by radiomics features performs poorly on the independent testing set, while temporal features showed surprising performance with only five time sequence features; moreover, fusion features have high robustness, producing an AUC of 0.942 and an ACC of 0.889 in the diagnosis. For AVM grading, the group with temporal features obtained an AUC of 0.871 and an ACC of 0.840, which were better than the one without temporal features. The results can be understood in the way that radiomics features represent static image features. and temporal features represent effective temporal features. Combined features integrate changes in the DSA series with radiomics features that can describe DSA videos more completely.

Several limitations should be noted. First, samples should be excluded when the nidus is too large that it covers the vascular structures because it will affect the credibility of temporal features extracted by the detection model. Second, multiposition images were not included to establish the DSA radiomics model. Although many studies have reported that multimodality images are helpful for classification tasks, multiposition images are not available for all included patients in the current study (38–40). Therefore, we select the anteroposterior position for the analysis to diminish the selection bias. Finally, more samples should be collected to provide a more convincing result.

## Conclusion

DSA videos provide an important basis for the diagnosis of cerebrovascular disease and provide a reference plan for surgical treatment, which is of great significance for the study of cerebrovascular disease. Our results suggest that temporal features obtained from DSA videos are representative and highly correlated with real-time medical images classification. DSA radiomics features combined with temporal features provide better performance in AVM analysis with high ACC.

However, DSA is a two-dimensional image that cannot describe blood vessels' shape or the blood flow rate. In the future, by combining CTA and DSA videos, more comprehensive modeling of cerebral blood vessels can be carried out. The influence of drainage veins and supply veins on the size of AVM can also be analyzed.

## Data Availability Statement

The raw data supporting the conclusions of this article will be made available by the authors, without undue reservation.

## Ethics Statement

The studies involving human participants were reviewed and approved by Huashan Hospital affiliated to Fudan University. Written informed consent to participate in this study was provided by the participants' legal guardian/next of kin.

## Author Contributions

KS and WX collected all the data and carried out the experiments. KS and WX wrote the manuscript with support from GW, YX, and YL. JY and YG helped supervise the project. All authors contributed to the article and approved the submitted version.

## Conflict of Interest

The authors declare that the research was conducted in the absence of any commercial or financial relationships that could be construed as a potential conflict of interest.
